# The effect of elevated muscle pain on neuromuscular fatigue during exercise

**DOI:** 10.1007/s00421-021-04814-1

**Published:** 2021-09-29

**Authors:** Ryan Norbury, Samuel A. Smith, Mark Burnley, Megan Judge, Alexis R. Mauger

**Affiliations:** Endurance Research Group, School of Sports and Exercise Sciences, University of Kent, Chipperfield Building, Canterbury Campus, Canterbury, CT2 7NZ Kent UK

**Keywords:** Muscle pain, Neuromuscular fatigue, Isometric performance, Central fatigue, Transcranial magnetic stimulation

## Abstract

**Purpose:**

Muscle pain can impair exercise performance but the mechanisms for this are unknown. This study examined the effects of muscle pain on neuromuscular fatigue during an endurance task.

**Methods:**

On separate visits, twelve participants completed an isometric time-to-task failure (TTF) exercise of the right knee extensors at ~ 20% of maximum force following an intramuscular injection of isotonic saline (CTRL) or hypertonic saline (HYP) into the vastus lateralis. Measures of neuromuscular fatigue were taken before, during and after the TTF using transcranial magnetic stimulation (TMS) and peripheral nerve stimulation.

**Results:**

The mean pain intensity was 57 ± 10 in HYP compared to 38 ± 18 in CTRL (*P* < 0.001). TTF was reduced in HYP (4.36 ± 0.88 min) compared to CTRL (5.20 ± 0.39 min) (*P* = 0.003). Maximum voluntary force was 12% lower at minute 1 (*P* = 0.003) and 11% lower at minute 2 in HYP (*P* = 0.013) compared to CTRL. Voluntary activation was 4% lower at minute 1 in HYP compared to CTRL (*P* = 0.006) but not at any other time point (all *P* > 0.05). The TMS silent period was 9% longer at 100 s during the TTF in HYP compared to CTRL (*P* = 0.026).

**Conclusion:**

Muscle pain reduces exercise performance through the excacerbation of neuromuscular fatigue that is central in origin. This appears to be from inhibitory feedback from group III/IV nociceptors which acts to reduce central motor output.

## Introduction

Exercise requires repeated or sustained muscular contractions and can cause a progressive decline in the force-generating capacity of a muscle, known as exercise-induced fatigue (Gandevia 2001). The aetiology of exercise-induced fatigue can be central (changes at the spinal or supraspinal level) and/or peripheral (changes at or distal to the neuromuscular junction) in origin (Bigland Ritchie et al. 1978; Kent-Braun 1999) but most exercise appears to encompass both types of fatigue in a feedback–feedforward system to regulate exercise tolerance (Hureau et al. 2018).

Strenuous exercise is usually accompanied by exercise-induced pain. Pain can be defined as an unpleasant sensory and emotional experience associated with, or resembling that associated with, actual or potential tissue damage (Raja et al. 2020). The naturally occurring and non-damaging exertional pain accompanying strenuous exercise (exercise-induced pain) can be described as “aching” or “cramping” and increases as a function of time/exercise intensity (Cook et al. 1997; Smith et al. 2020). The feeling of exercise-induced pain arises from the accumulation of noxious biochemicals, reduced muscle pH and increases in intramuscular pressure which consequently stimulates group III/IV nociceptive afferents (O’Connor and Cook 1999; Mense 2008). Since exercise-induced pain and exercise intensity (and consequently the development of fatigue) are associated, it may be possible that exercise-induced pain contributes to the fatigue process, however this is not known.

Previous work has found that in combination with traditional physiological parameters (e.g. lactate threshold), pain tolerance (i.e. the maximum level of perceived pain someone can tolerate) can partially predict cycling time-trial performance (Astokorki and Mauger 2017a) and that reducing muscle pain through the ingestion of acetaminophen results in an improvement in endurance performance (Mauger et al. 2010; Foster et al. 2014; Astokorki and Mauger 2017b; Morgan et al. 2019). Conversely, elevating muscle pain through the intramuscular injection of hypertonic saline has been shown to reduce isometric TTF performance (Graven-Nielsen et al. 1997b; Ciubotariu et al. 2004; Smith et al. 2020) and maximum muscle strength (Graven-Nielsen et al. 2002; Slater et al. 2003; Khan et al. 2011). The mechanisms which underpin these changes are suggested to be centrally mediated (Le Pera et al. 2001; Schabrun and Hodges 2012) but the fatiguing effect of pain during exercise is unclear. Additionally, the experience of muscle pain may reduce endurance performance by acting as an aversive stimulus which causes a voluntary disengagement from exercise or reduction in exercise intensity. On the other hand, muscle pain may independently cause fatigue by altering motor unit recruitment thresholds/firing rates or reducing central motor drive and act on a physiological, unconscious basis (i.e., the nociceptive component).

Recently, Smith et al. (2020) induced muscle pain using an intramuscular injection of hypertonic saline during submaximal isometric knee extensor exercise. They found that this produced a similar pain quality to exercise-induced pain and allowed the authors to decouple the pain-intensity relationship during knee extensor exercise. The increased muscle pain caused a mean decrease of 26% in endurance time, despite a similar end-exercise maximum voluntary torque, which suggests that fatigue occurred more rapidly when pain was exacerbated.

The use of peripheral nerve stimulation allows for the measurement of peripheral changes in muscle function (e.g. resting twitch amplitude) as well as central changes in voluntary activation (via the interpolated twitch technique). Transcranial magnetic stimulation (TMS) allows for the non-invasive quantification of corticospinal excitability and inhibition during exercise and in combination would provide novel information on the development of neuromuscular fatigue in response to elevated muscle pain. Consequently, these methods allow us to further understand the mechanisms of how muscle pain may act to limit endurance performance as opposed to isolated measures of motor function that have previously been explored (e.g. Le Pera et al. 2001; Khan et al. 2011).

Therefore, the purpose of this study was to perform an isometric TTF of the knee extensors with elevated muscle pain from an intramuscular injection of hypertonic saline while simultaneously recording measures of neuromuscular fatigue to identify the mechanisms behind how muscle pain limits endurance performance. It was hypothesised that the intramuscular injection of hypertonic saline would decrease isometric TTF through an exacerbation of central fatigue (i.e., decreased voluntary activation).

## Methods

### Participants

Twelve healthy and recreationally active individuals (two female) with a mean ± SD age 26.6 ± 3.9 years, height: 175 ± 8.2 cm, body mass: 72.2 ± 11.7 kg volunteered to take part in the study. All participants had no lower-limb injury within the past three months, were not taking medication for the treatment of pain or had any pain related conditions. Participants were also screened for any contraindications to TMS. All participants provided written informed consent before testing. The study was approved by the University of Kent SSES Research Ethics Advisory Group (Prop 30_2018_2019) and was conducted in alignment with the declaration of Helsinki.

### Experimental protocol

**Fig. 1:**
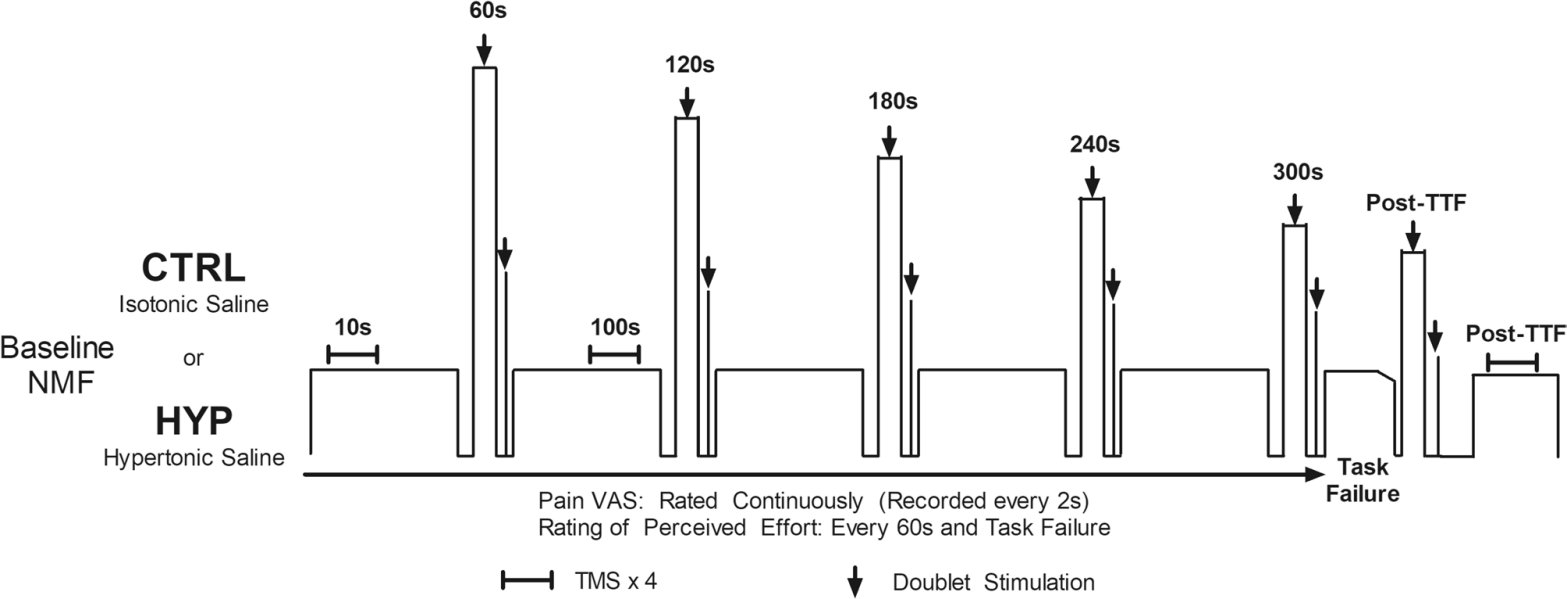
Schematic of the experimental visits CTRL and HYP. *EMG* electromyography, *VL* vastus lateralis, *VM* vastus medialis, *BF* biceps femoris, *VAS* visual analogue scale, *NMF* neuromuscular function

Participants visited the laboratory on four occasions separated by a minimum of 48 h between visits 1 and 2 and at least 7 days between visits 3 and 4. Participants performed the experiment at a similar time of day (± 1.5 h) and avoided strenuous physical activity 48 h, caffeine 4 h, alcohol 24 h and analgesics 6 h prior to testing. In visit one, participants were familiarised with measures of neuromuscular function (see neuromuscular function testing), questionnaires, perceptual measures, the isometric TTF exercise and the intramuscular injection of hypertonic saline if they had not received one before. Visit two composed of a second familiarisation of the isometric exercise task where the intensity (%MVC) was adjusted from the first visit if the TTF was not within 4–6 min. This was to ensure that the isometric time-to-task failure coincided with the typical pain duration from the intramuscular injection of hypertonic saline into the vastus lateralis (VL) (Smith et al. 2020). Visits three and four were experimental visits (Fig. 1) completed in a randomised order. Participants arrived at the laboratory and completed the positive and negative affect schedule (PANAS) and pain expectation/pain-coping confidence. They then underwent baseline measures of neuromuscular function involving peripheral neve stimulation and single pulse TMS during isometric contractions of the right knee extensors. Participants then waited 10 min before receiving an intramuscular injection of 1 mL of isotonic saline (0.9%) or hypertonic saline (5.85%) in the muscle belly of the VL. The isotonic saline condition served as a non-painful injection matched control (CTRL) while the hypertonic saline caused acute muscle pain (HYP). Immediately after the injection, participants began the submaximal isometric TTF protocol with intermittent measures of peripheral nerve stimulation and TMS while providing measures of pain and RPE until task failure, where post-exercise measures of neuromuscular fatigue were performed along with the pain catastrophizing scale (Edwards et al. 2006) and McGill long form pain questionnaire (Melzack 1975).

### Equipment and procedures

#### Experimental muscle pain

A single bolus of 1 mL hypertonic saline (5.85% NaCl) was injected in the VL (the middle third of the muscle belly) of the right leg to induce muscle pain. The site was cleaned with an alcohol swab and then the saline was manually injected using a 3 mL Luer-Lok syringe (BD, New Jersey, USA) connected to a 1.5 inch 25-gauge hypodermic needle (SurGuard2, Terumo, Japan) over a 20 s window (5 s pause after the insertion, a 10 s infusion period, followed by 5 s pause before needle withdrawal). An identical injection protocol was performed with the isotonic saline (CTRL condition).

#### Exercise protocol

The exercise protocol was a semi-constant submaximal isometric TTF of the right knee extensors at an individualised intensity to cause TTF within 4–6 min in CTRL. The mean intensity for the participants was 20% of maximum voluntary force (MVF), but this ranged from 13 to 25% of MVF. Three seconds before the end of each minute in the TTF exercise, participants were instructed to relax and prepare to perform an MVC with superimposed doublet and subsequently relax for 3 s while a resting doublet was delivered. Four TMS pulses and a single peripheral nerve stimulation was delivered during the submaximal contraction phase of the TTF task at 10 s and 100 s. Participants were encouraged to go for as long as possible until they were unable to maintain the target for three consecutive seconds or voluntarily withdrew from the task. Participants also continuously rated their pain and provided RPE every minute and at task failure. A schematic of the experimental protocol can be seen in Fig. 1.

#### Mechanical recordings

Participants were strapped into a custom-built isometric chair with a hip and knee angle of 90° (0° being full extension). Straps secured the participant around the torso to prevent any extraneous movement and a non-compliant strap was secured 2 cm above the malleoli which was connected to a transducer to measure isometric force of the knee extensors. The transducer was connected to a signal amplifier (DA100c, Biopac Systems Inc, California, USA) and data acquisition module (MP150, Biopac Systems Inc, California, USA) and sampled at a frequency of 1.25 kHz in compatible software (Acqknowledge 5.0, Biopac Systems, California, USA). Force traces providing instantaneous feedback were displayed on a computer screen in view of the participant.

#### Electromyography (EMG)

Bipolar surface electromyography was used to record activity of the VL, vastus medialis (VM) and biceps femoris (BF) with 37.5 mm × 37.5 mm Ag/AgCl electrodes (Whitesensor 4831Q, Ambu Ltd, Denmark) at an inter electrode distance of 37.5 mm. The VL electrodes recorded evoked responses from TMS and peripheral nerve stimulation and voluntary muscle activity whereas the VM was used to assess changes in synergist activity in response to muscle pain. The BF measures were used to check and minimise antagonist motor-evoked potential amplitudes.

The electrode location was on the muscle belly proximal to the knee and parallel to the fibres of the muscle for the VL and VM while the BF was placed on the muscle belly 50% of the distance between the ischial tuberosity and the lateral epicondyle of the tibia. Each site was shaved, abraded and cleaned to reduce impedance and the electrode locations were marked for replication in subsequent visits. All EMG data were recorded continuously at a frequency of 2.5 kHz and amplified (gain 1000 for VL, 2000 for VM and BF) with a signal amplifier (EMG2-R, Biopac Systems, California, USA and EMG100c, Biopac Systems, California, USA) before being band pass-filtered (10–500 Hz) and recorded onto compatible software (Acqknowledge v5.0, Biopac systems, California, USA).

#### Peripheral nerve stimulation

An electrical stimulator (DS7r, Digitimer, Hertfordshire, UK) (maximum voltage = 400 v) capable of delivering a single square wave pulse was used for peripheral nerve stimulation. The anode was an adhesive electrode (100 mm × 50 mm; Phoenix Healthcare Products Ltd, Nottingham, UK) which was secured at the gluteal fold. Initially, the cathode was a motor-point pen (Motor Point Pen; Compex; DJO Global, Guildford, UK) which was placed over the femoral nerve. The motor-point pen was used to identify the precise location which evoked the largest twitch force and compound muscle action potential (M-wave) peak-to-peak amplitude. A 32 × 32 mm electrode was attached to this site (Nessler Medizintechnik, Innsbruck, Austria) to ensure the same area was stimulated for all subsequent stimulations. This process was repeated on every experimental visit. To determine the intensity required to achieve supramaximal stimulation, 20 mA stepwise increments in stimulation intensity were delivered from 100 mA until a plateau in twitch force and M-Wave amplitude was observed. An additional 30% was added to ensure supramaximal stimulation (*M*_max_). This intensity was used throughout the rest of the respective trial. Doublets were delivered as 100 Hz paired stimuli (1 ms pulse duration) for the assessment of central and peripheral fatigue (see below data analysis), whereas single stimulus (2 ms pulse duration) was delivered for the normalisation of motor-evoked potentials.

#### Transcranial magnetic stimulation

Single-pulse TMS was delivered with a magnetic stimulator (Magstim 200^2^, The Magstim Company Ltd, Carmarthenshire, UK) via a double cone coil (110 mm diameter) delivering a posterior–anterior current which was placed over the motor cortex to assess corticospinal excitability of the right quadriceps. Initially the participant’s vertex was marked as the midpoint between the nasal-inion and the tragus. The coil was initially placed 2 cm laterally to this position. Stimuli were superimposed over a submaximal contraction (same as target force for subsequent exercise ~ 20% of maximum force) of the knee extensors at 50% of maximal stimulator output until the hotspot (the location which provided the greatest peak-to-peak EMG amplitude of the motor-evoked potential [MEP_AMP_] in the VL while minimising the MEP_AMP_ of the BF (to ensure optimal coil placement) was found. This location was marked onto a skin-tight hat the participant was wearing. The participant also wore a cervical collar to prevent excessive movement of the head. Subsequent TMS pulses were delivered in trains of four stimuli separated by approximately 3 s which were superimposed over the submaximal knee extensor contraction. Trains of stimuli were separated by approximately 20 s during baseline. Stepwise increments in the stimulator intensity of 5% were used until a plateau in the average of the four MEPs was reached (< 5% increase). This was 67 ± 5% of maximum stimulator output in CTRL and 66 ± 7% in HYP. Each train of TMS pulses was accompanied by the delivery of a single peripheral nerve stimulation to acquire MEP/*M*_max_ ratio.

#### Perceptual measures

To assess the quantity of pain, an electronic visual analogue scale displayed the pain perception scale (Cook et al. 1997) and allowed participants to continuously rate their muscle pain. The device automatically recorded a pain reading every 2 s based on the position of the slider marker and recorded the data on an SD card. The scale ranged from 0 which corresponded to ‘no pain at all’ to 100 which corresponded to ‘extremely intense pain (almost unbearable)’. Participants were instructed to anchor the upper pain ratings to the worst exercise-induced pain they had previously experienced (Astokorki and Mauger 2017b). Rating of perceived effort (RPE) was recorded on the 6–20 point scale (Borg 1998) to avoid participants conflating pain and effort ratings. Instructions were also given to participants to exclusively rate their effort based on the ‘effort to drive the limb’ with a rating of 20 anchored to the level of drive given during the maximum voluntary contraction performed prior (Pageaux 2016).

#### Questionnaires

Before each experimental visit, the PANAS (Watson et al. 1988) was administered to confirm participants arrived at the lab in a similar psychologoical state. Additionally, pain expectation from 0 to 10 (0 = ‘no pain’ and 10 = ‘worst possible pain’) and perceived pain-coping ability (0 = ‘not confident at all’ and 10 = ‘completely confident’) were recorded. The situation-specific pain catastrophizing scale (Edwards et al. 2006) and the long form McGill pain questionnaire (Melzack 1975) were administered immediately post exercise.

#### Neuromuscular function testing

For baseline measures of neuromuscular function, participants initially performed a warmup consisting of ten contractions at 50% of perceived maximum effort (3 s contracting, 3 s relaxing). This was followed by four maximum voluntary contractions of 4 s in duration separated by 2 min of rest. On the third and fourth MVC, a superimposed doublet was delivered once peak force was reached and a resting potentiated doublet was delivered within 5 s of the end of the MVC. Twelve TMS stimuli were delivered during twelve submaximal contractions (3 sets of 4 contractions) at the target force of the subsequent exercise. One single peripheral nerve stimulation was delivered during a contraction after the final contraction with TMS. Post exercise (within 10 s), a single MVC with peripheral neve stimulation was delivered followed by four submaximal contractions superimposed with TMS and one contraction superimposed with single peripheral nerve stimulation to measure corticospinal excitability and inhibition.

### Data analysis

The baseline neuromuscular variables were calculated as the mean raw value and the raw value was taken for each measure during every minute. MVF and doublet amplitude were recorded as the peak instantaneous force achieved. Voluntary activation, a measure of central fatigue, was calculated using the interpolated twitch technique with the Strojnik and Komi (1998) correction applied where necessary, with VA calculated as:

$$100\; - {\text{ SI Doublet}} \times \frac{{\left( {{\text{force before SI doublet/peakforce}}} \right)}}{{{\text{resting potentiated doublet}}}} \times 100.$$

The average MEP peak-to-peak amplitude was normalised to the most proximal peak-to-peak amplitude of the M-Wave to get MEP/*M*_max_. The TMS silent period was determined as the duration from the point of stimulation (i.e. stimulation artefact) until the return of the EMG signal which was visually inspected by the same investigator. The root mean square (RMS) of the EMG waveform was calculated offline in software (Acqknowledge V5.0; Biopac systems Inc, California, USA) using a 100 ms time constant. The mean 500 ms of the RMS (250 ms either side of peak force) was analysed for MVCs and the mean 20 s of data was analysed at the beginning of each minute and before task failure of the exercise task and was normalised to MVC EMG amplitude. The ΔMVF, ΔVAL, ΔDoublet, ΔSilent period/ΔTime were calculated as the change in value from pre- to post-exercise divided by the TTF as an indicator of the rate of fatigue development.

Pain data were taken as the VAS recorded at every 20 s and at task failure.

### Statistical analysis

All data are presented as mean ± SD or a mean and interquartile range when not normally distributed. Data were analysed in JAMOVI 1.0.7.0. (The Jamovi Project, 2020). Data were initially checked for normality with the Shapiro–Wilk test and sphericity with the Mauchly test. If these assumptions were violated, data were analysed with a non-parametric test or Greenhouse–Geiser corrected, respectively. A paired samples *t* test was used to compare TTF between CTRL and HYP. A 2 × 4 repeated measures ANOVA (condition × time) was used to analyse neuromuscular variables at baseline, minute one, two (or 10 s and 100 s for TMS data) and task failure. A 2 × 8 repeated measures analysis of variance (ANOVA) was used to analyse pain VAS data. Follow-up paired samples t tests were used to determine differences between conditions at different time points and were Bonferroni–Holm-corrected where appropriate (Holm 1979). Paired samples *t* tests were used for differences in TTF and the ΔMVF, ΔVAL, ΔDoublet, ΔSP/ΔTime which were Bonferroni-corrected. Intraclass correlation coefficients (2,1) were calculated and presented as point estimate and 95% confidence interval for doublet amplitude between CTRL ansd HYP at minute one, two and task failure for confirmation of similarity.

95% confidence intervals, Cohen’s *d* effect sizes (Cohen 1992) and partial eta-squared (${\upeta }_{\mathrm{p}}^{2}$) were reported where appropriate. A Pearson correlations matrix was used to examine the relationship between changes in pain at minute 1 between conditions against change in neuromuscular variables between conditions at minute 1, and was Bonferroni-corrected.

## Results

### Time to task failure

**Fig. 2:**
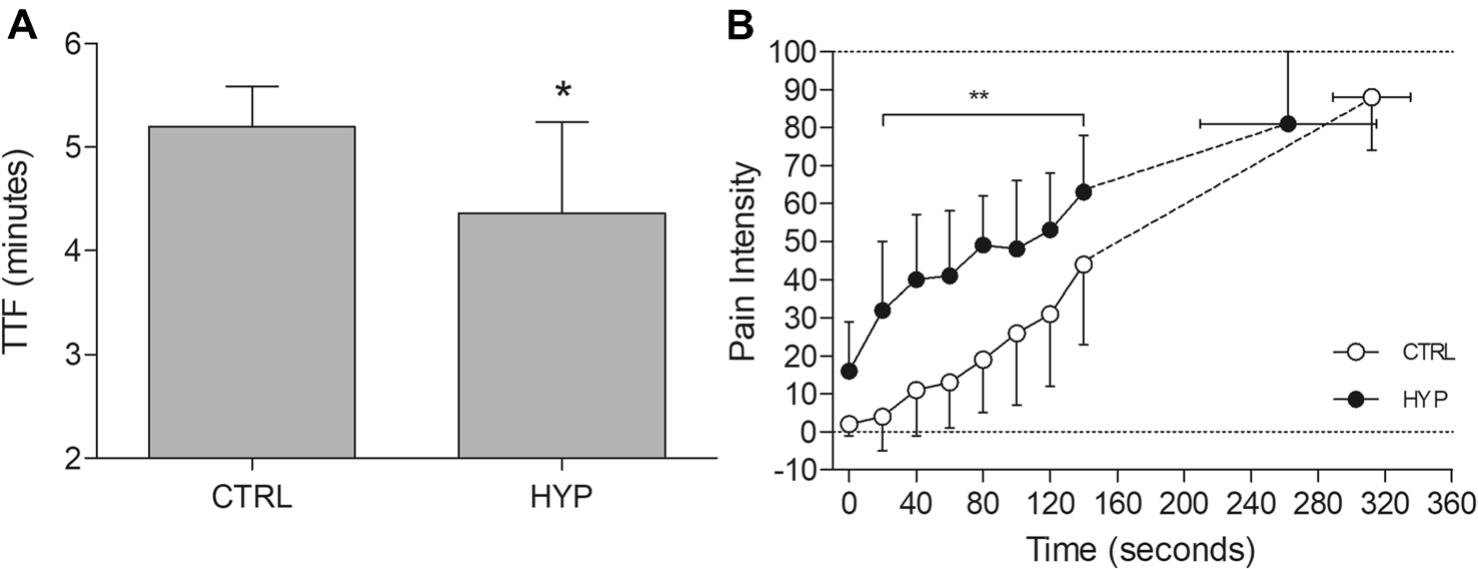
**A** TTF of the isometric endurance task. Data presented as mean ± SD and individual data. *Denoted significantly different from CTRL (*P* < 0.05). **B** Pain VAS data through the isometric TTF. Data presented as mean ± SD. **Denotes significantly different from CTRL (*P* < 0.001)

There was a 16.2% shorter TTF in HYP (4.36 ± 0.88 min) compared to CTRL (5.20 ± 0.39 min) (mean difference = 0.84 min, 95% CI [0.34, 1.33 min], *t*_11_ = 3.728, *P* = 0.003, *dz* = 1.08) (Fig. 2).

### Pain intensity and pain quality

Prior to each experimental visit, there was no difference in pain expectation (*P* = 0.602) or pain-coping confidence (Wilcoxon *P* = 1.000). The mean pain intensity, matched for exercise time, was greater in HYP (57 ± 10) compared to CTRL (38 ± 18) (mean difference = 19, 95% CI [11, 28], *t*_11_ = 5.18, *P* < 0.001, *dz* = 1.50). When also matched for exercise time, peak pain was greater in HYP (94.5 [75.8–99.3]) compared to CTRL (85.5 [55.8–99.0]) (Wilcoxon *P* = 0.047). For pain intensity throughout the TTF, there was a condition × time interaction (*F*_3.42, 37.59_ = 10.7, *P* < 0.001, ${\upeta }_{\mathrm{p}}^{2}$ = 0.493) (Fig. 2). Pain intensity was elevated from 20 to 140 s (all *P* < 0.001) in HYP compared to CTRL but was not different between conditions at 0 s (*P* = 0.142) or at task failure (*P* = 1.000) (Fig. 2). For pain quality assessed by the McGill long form questionnaire, Cramping (50%), Aching (58%), Tiring (58%) and Intense (50%) were the most common words selected in CTRL whereas in HYP, Cramping (50%), Aching (42%), Grueling (42%), Intense (67%) were most selected. No difference was seen in the total pain rating (*P* = 0.466) or the sensory (*P* = 0.686), affective (*P* = 0.515), evaluative (Wilcoxon *P* = 0.269) or miscellaneous (*P* = 0.160) dimensions of pain.

### Maximum voluntary force (MVF)

**Fig. 3:**
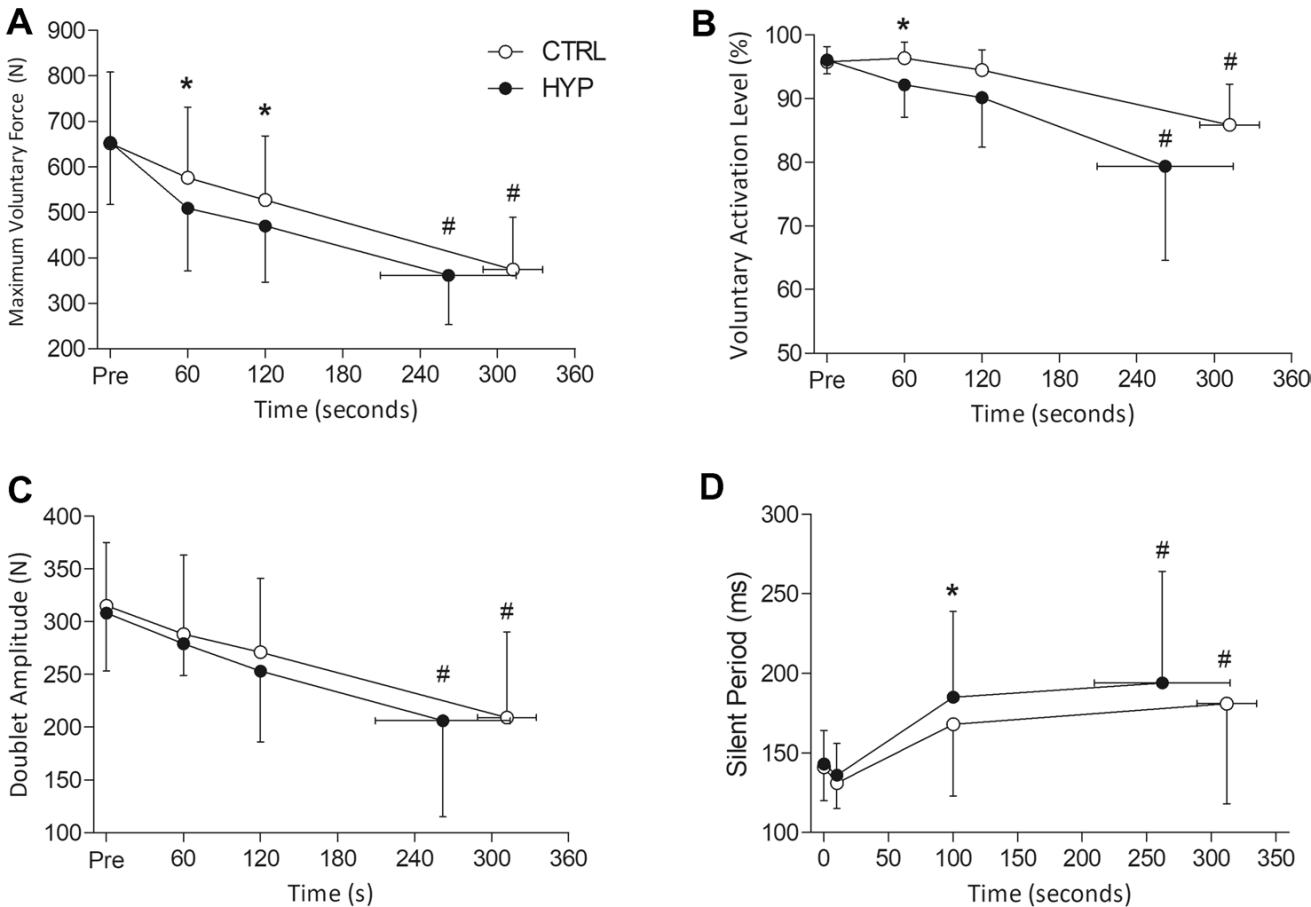
Neuromuscular fatigue variables at each minute of the isometric TTF. **A** Maximum voluntary force. **B** Voluntary activation level. **C** Doublet Amplitude. **D** Silent Period during the MVCs. *Denotes significantly different from CTRL (*P* < 0.05). ^#^Denotes significantly different from baseline (*P* < 0.05)

For MVF, there was a condition × time interaction (*F*_1.77, 19.43_ = 6.81, *P* = 0.007, ${\upeta }_{\mathrm{p}}^{2}$ = 0.382). Subsequent post hoc tests revealed that MVF decreased by 43% (mean difference = 278 N, 95% CI [218, 338 N], *t*_11_ = 14.09, *P* < 0.001, *dz* = 2.96) and 45% (mean difference = 293 N, 95% CI [244, 342 N], *t*_11_ = 14.85, *P* < 0.001, *dz* = 3.78) in CTRL and HYP, respectively, with no difference between conditions (*P* > 0.999). However, during the exercise task, MVF was lower at minute 1 in HYP (509 ± 139 N) compared to CTRL (577 ± 155 N) (mean difference = 68 N, 95% CI [26, 109 N], *t*_11_ = 4.001, *P* = 0.003, *dz* = 1.02). Similarly, MVF at minute 2 was lower in HYP (470 ± 124 N) compared to CTRL (527 ± 141 N) (mean difference = 56 N, 95% CI [10, 102 N], *t*_11_ = 3.334, *P* = 0.013, *dz* = 0.78) (Fig. 3.). The change in ΔMVF/ΔTime was greater in HYP than in CTRL (Wilcoxon *P* = 0.015) (CTRL = 52 [43–63] N min^−1^, HYP = 67 [56–81] N min^−1^).

### Voluntary activation (VA)

No interaction effect was observed for VA (*F*_1.56, 17.18_ = 1.34, *P* = 0.282, ${\upeta }_{\mathrm{p}}^{2}$ = 0.108). However, there was a main effect of condition (*F*_1.11,_ = 7.60, *P* = 0.019, ${\upeta }_{\mathrm{p}}^{2}$ = 0.409). Post hoc tests revealed that VA was lower in HYP (92.2 ± 5.1%) than CTRL (96.4 ± 2.5%) at minute 1 (mean difference = 4.2%, 95% CI [1.44, 6.9%], *t*_11_ = 3.36, *P* = 0.006, *dz* = 0.97) but was not different at minute 2 (mean difference = 4.3%, 95% CI [− 0.2%, 8.8%], *t*_11_ = 2.08, *P* = 0.061, *dz* = 0.60) or at task failure (mean difference = 6.5%, 95% CI [− 0.5%, 13.5%], *t*_11_ = 2.04, *P* = 0.066, *dz* = 0.59). There was also a main effect of time for VA (*F*_1.45, 15.91_ = 17.31, *P* < 0.001, ${\upeta }_{\mathrm{p}}^{2}$ = 0.611). VA decreased from 95.8 to 85.9% in CTRL (mean difference = 9.9%, 95% CI [6.1, 13.8%], *t*_11_ = 4.047, *P* = 0.003, *dz* = 1.64) and from 96.1% to 79.4% in HYP (mean difference = 16.69%, 95% CI [7.8, 25.5%], *t*_11_ = 6.807, *P* < 0.001, *dz* = 1.20). There was a greater ΔVAL/ΔTime in HYP (3.9 ± 3.0% min^−1^) compared to CTRL (1.9 ± 1.2% min^−1^) (*P* = 0.036).

### Doublet amplitude and *M*_max_

For doublet amplitude, there was no condition x time interaction (*F*_1.48, 16.24_ = 0.346, *P* = 0.649, ${\upeta }_{\mathrm{p}}^{2}$ = 0.030) or main effect of condition (*F*_1, 11_ = 1.578, *P* = 0.235, ${\upeta }_{\mathrm{p}}^{2}$ = 0.125). However, there was a main effect of time (*F*_1.07, 11.80_ = 22.136, *P* < 0.001, ${\upeta }_{\mathrm{p}}^{2}$ = 0.668). Doublet amplitude decreased by 34% in CTRL (mean difference = 106 N, 95% CI [64, 148 N], *t*_11_ = 7.725, *P* < 0.001, *dz* = 1.60) and by 33% in HYP (mean difference = 103 N, 95% CI [57, 149 N], *t*_11_ = 7.510, *P* < 0.001, *dz* = 1.43) (Fig. 3). There was no difference in the ΔDoublet/ΔTime (*P* = 0.218). Intraclass correlation coefficients for doublet amplitude were 0.935 (0.795–0.981), 0.948 (0.836–0.985) and 0.944 (0.819–0.984). For *M*_max_, there was no condition × time interaction (*F*_3, 33_ = 1.360, *P* = 0.272, ${\upeta }_{\mathrm{p}}^{2}$ = 0.110) or main effect of condition (*F*_1, 11_ = 0.074, *P* = 0.790, ${\upeta }_{\mathrm{p}}^{2}$ = 0.007) and time (*F*_1.90, 20.91_ = 3.26, *P* = 0.061, ${\upeta }_{\mathrm{p}}^{2}$ = 0.229).

### MEP/*M*_max_

For MEP_AMP_, no condition × time interaction was observed (*F*_1.69, 18.54_ = 0.370, *P* = 0.660, ${\upeta }_{\mathrm{p}}^{2}$ = 0.033) or main effect of condition *F*_1, 11_ = 2.411, *P* = 0.149, ${\upeta }_{\mathrm{p}}^{2}$ = 0.180). There was a main effect of time (*F*_3, 33_ = 3.942, *P* = 0.017, ${\upeta }_{\mathrm{p}}^{2}$ = 0.264) for an increase in MEP/*M*_max_ but subsequent post hoc tests with a Holm–Bonferroni-correction revealed no significant differences.

### Silent period

There was no condition × time interaction for silent period (*F*_2.08, 22.85_ = 1.84, *P* = 0.181, ${\upeta }_{\mathrm{p}}^{2}$ = 0.143). However, there was a main effect of time (*F*_1.24, 13.66_ = 10.56, *P* = 0.004, ${\upeta }_{\mathrm{p}}^{2}$ = 0.490) and condition (*F*_1, 11_ = 6.47, *P* = 0.027, ${\upeta }_{\mathrm{p}}^{2}$ = 0.370). Silent period increased by 28% in CTRL (mean difference = 40 ms, 95% CI [9, 72 ms], *t*_11_ = 3.368, *P* = 0.031, *dz* = 0.81) and by 36% in HYP (mean difference = 51 ms, 95% CI [15, 87 ms], *t*_11_ = 4.304, *P* = 0.003, *dz* = 0.91) but was not different between conditions (mean difference = 13 ms, 95% CI [− 5, 31 ms], *t*_11_ = 1.60, *P* = 0.138, *dz* = 0.46). A longer silent period was observed at the 100 s time point (mean difference = 17 ms, 95% CI [2, 31 ms], *t*_11_ = 2.57, *P* = 0.026, *dz* = 0.74), but not at 10 s (mean difference = 5 ms, 95% CI [− 3, 14 ms], *t*_11_ = 1.42, *P* = 0.183, *dz* = 0.41) (Fig. 3).

### Electromyography

#### Vastus lateralis

**Fig. 4:**
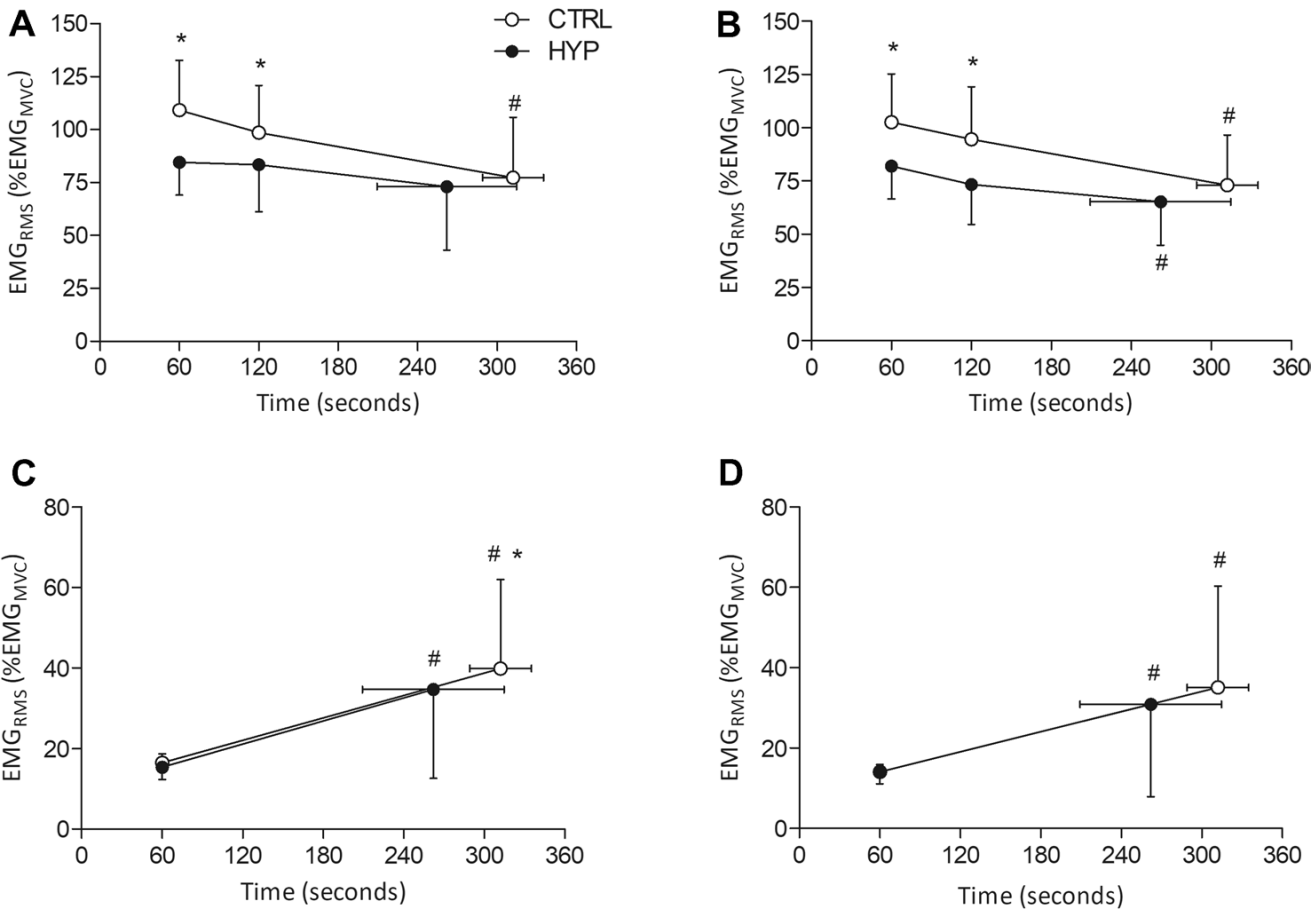
Root mean square electromyographic recordings during MVCs and the submaximal isometric TTF. **A** Vastus lateralis MVC EMG amplitude. **B** Vastus medialis MVC EMG amplitude. **C** Vastus lateralis isometric TTF EMG amplitude. **D** Vastus medialis isometric TTF EMG amplitude. *Denotes significantly different from CTRL (*P* < 0.05). ^#^Denotes significantly different from minute 1 (*P* < 0.05)

For EMG_RMS_ amplitude of the VL during MVCs, there was a condition × time interaction (*F*_2, 22_ = 4.74, *P* = 0.019, ${\upeta }_{\mathrm{p}}^{2}$ = 0.301). EMG_RMS_ was lower at minute 1 (mean difference = 24.8%, 95% CI [12.6, 37.1%], *t*_11_ = 4.978, *P* < 0.001, *dz* = 1.29) and minute 2 (mean difference = 15.1%, 95% CI [4.0, 26.1%], *t*_11_ = 3.024, *P* = 0.044, *dz* = 0.87) in HYP compared to CTRL. No difference was seen at task failure (mean difference = 4.4%, 95% CI [− 5.1, 13.8%], *t*_11_ = 0.877, *P* = 1.000, *dz* = 0.29). EMG_RMS_ decreased in CTRL from minute 1 to task failure (mean difference = 31.9%, 95% CI [14.4, 49.4%], *t*_11_ = 5.180, *P* < 0.001, *dz* = 1.16) but not it HYP (*P* = 0.500). For EMG amplitude during the submaximal TTF, there was a condition × time interaction (*F*_1, 11_ = 5.018, *P* = 0.047, ${\upeta }_{\mathrm{p}}^{2}$ = 0.313). EMG_RMS_ was not different at minute 1 (mean difference = 1.1%, 95% CI [-1, 3.1%], *t*_11_ = 0.743, *P* = 0.465, *dz* = 0.33). EMG_RMS_ increased in amplitude at task failure for both conditions, however, EMG_RMS_ was lower in HYP compared to CTRL (mean difference = 5.2%, 95% CI [1.3, 9.2%], *t*_11_ = 3.795, *P* = 0.011, *dz* = 0.84) (Fig. 4).

#### Vastus medialis

For EMG_RMS_ amplitude of the VM during MVCs, there was no condition × time interaction (*F*_2, 22_ = 3.20, *P* = 0.060, ${\upeta }_{\mathrm{p}}^{2}$ = 0.225). However, there was a main effect of condition (*F*_1, 11_ = 8.58, *P* = 0.014, ${\upeta }_{\mathrm{p}}^{2}$ = 0.225). MVC EMG_RMS_ amplitude was lower at minute 1 (mean difference = 20.7%, 95% CI [5.7, 35.7%], *t*_11_ = 3.04, *P* = 0.033, *dz* = 0.88) and minute 2 (mean difference = 21.2%, 95% CI [3.8, 38.8%], *t*_11_ = 2.68, *P* = 0.042, *dz* = 0.77) but not different at the task failure MVC (mean difference = 8.0%, 95% CI [− 2.5, 18.5%], *t*_11_ = 1.67, *P* = 0.123, *dz* = 0.48). There was also a main effect of time (*F*_1.253, 13.779_ = 15.49, *P* < 0.001, ${\upeta }_{\mathrm{p}}^{2}$ = 0.585). MVC EMG_RMS_ decreased from minute 1 to task failure in CTRL (mean difference = 29.4%, 95% CI [13.5, 45.4%], *t*_11_ = 5.17, *P* < 0.001, *dz* = 1.17) and in HYP (mean difference = 16.7%, 95% CI [7.8, 25.6%], *t*_11_ = 3.69, *P* = 0.004, *dz* = 1.20). For EMG_RMS_ amplitude during the submaximal TTF, there was no condition × time interaction (*F*_1, 11_ = 3.401, *P* = 0.092, ${\upeta }_{\mathrm{p}}^{2}$ = 0.236) or main effect of condition (*F*_1, 11_ = 2.355, *P* = 0.153, ${\upeta }_{\mathrm{p}}^{2}$ = 0.176). There was a main effect of time (*F*_1, 11_ = 8.705, *P* = 0.013, ${\upeta }_{\mathrm{p}}^{2}$ = 0.442). EMG_RMS_ increased from minute 1 to task failure in CTRL (mean difference = 21.0%, 95% CI [5.6, 36.3%], *t*_11_ = 3.005, *P* = 0.012, *dz* = 0.87) and in HYP (mean difference = 16.8%, 95% CI [3.6, 30.0%], *t*_11_ = 2.807, *P* = 0.017, *dz* = 0.81) (Fig. 4).

### Correlations

**Fig. 5:**
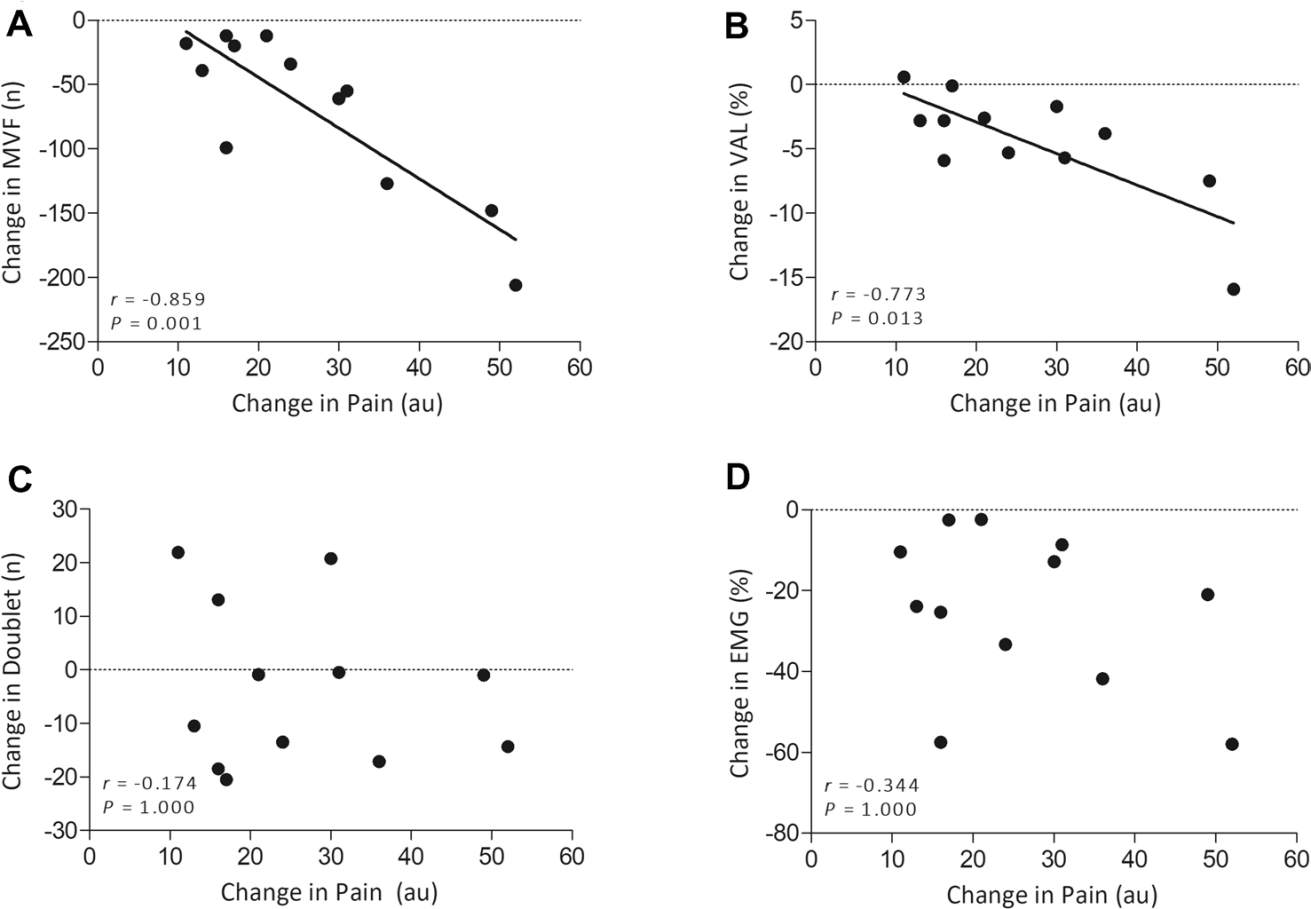
Pearson correlations between the change in pain between conditions for the first minute of the isometric TTF against the difference in the change in neuromuscular function variables at minute 1. **A** Maximum voluntary force. **B** Voluntary activation level. **C** Doublet amplitude. **D** EMG MVC amplitude of the VL

A Pearson correlation matrix with a Bonferroni correction revealed a significant negative relationship between the change in mean pain VAS from CTRL to HYP of minute 1, against the change in MVF (*r* = -0.859, *P* = 0.001) and VAL (*r* = − 0.773, *P* = 0.013) but not between doublet amplitude (*r* = − 0.174, *P* = 1.000) or MVC EMG amplitude (*r* = − 0.344, *P* = 1.000) between conditions (Fig. 5).

### Rating of perceived effort

For RPE, there was a condition × time interaction (*F*_2, 22_ = 12.6, *P* < 0.001, ${\upeta }_{\mathrm{p}}^{2}$ = 0.553). RPE increased over time but was greater at minute 1 in HYP (13 [12.4–14.0]) compared to CTRL (12 [11–12]) (Wilcoxon *P* = 0.008) and minute 2 (HYP = 15 [15–15.3], CTRL = 14 [13.4–14]) (Wilcoxon *P* = 0.024). No difference was seen at task failure (CTRL = 20 [20–20]), HYP = 20 [19.8–20]) (Wilcoxon *P* = 1.000).

### Pain catastrophising

There was no difference in the sum of pain catastrophising score (mean difference = 1.1, 95% CI [− 3.4, 1.1], *t*_11_ = 1.13, *P* = 0.281, *dz* = 0.33).

## Discussion

The novel findings of the study are twofold: (i) Increased muscle pain reduces endurance performance and maximal strength, (ii), these reductions in performance can be attributed to the excacerbation of central fatigue as seen by greater decreases in voluntary activation and a longer silent period in HYP compared to CTRL. Furthermore, similar decreases in evoked responses were achieved in a shorter time.

### Pain on isometric TTF

The intramuscular injection of hypertonic saline prior to a submaximal isometric TTF elevated leg muscle pain by 36% when conditions were matched for exercise time, and pain was particularly exacerbated within the first two minutes of exercise in HYP compared to CTRL. This increase in leg muscle pain, which was similar in quality to that of exercise-induced pain (i.e., no difference in McGill questionnaire ratings) resulted in a mean 16.2% (*dz* = 1.08) decrease in isometric TTF. These findings are similar to other studies which have investigated endurance performance in response to pain such as Graven-Nielsen et al (1997a, b) who saw a ~ 20% reduction in TTF during an isometric dorsiflexion at 80% of maximum torque when hypertonic saline was injected into the tibialis anterior and Smith et al. (2020) (*d* = 0.6) who injected hypertonic saline into the VL and performed an isometric TTF at 20% of maximum torque for the knee extensors. Conversely, no difference in TTF at 40% of maximum torque was observed when the biceps brachii were injected with hypertonic saline (Schulte et al. 2004). Variation in the TTF reducing effect is likely a product of the different muscle groups tested, chosen exercise intensity and volume/concentration of hypertonic saline used. A fixed volume of hypertonic saline (1 mL, 5.85%) is also likely to cause a varying pain response among individuals (Graven-Nielsen et al. 1997a) as there are likely differences in pain processing among participants (Fillingim 2017). This appeared to be the case in the present study as VAS ratings varied greatly (see Fig. 2b). This may explain some of the variability in changes in TTF in this study as some participants’ mean pain was only slightly greater in HYP compared to CTRL. Nevertheless, this study demonstrates a notable decrease in endurance performance when muscle pain is increased in a locomotor muscle which is functionally important for common endurance tasks (e.g., running or cycling).

### Pain and neuromuscular performance

Elevated muscle pain in HYP resulted in a decrease in maximum voluntary force at minute 1 and 2 during the TTF compared to CTRL. This decrease not only demonstrates the ability of pain to reduce maximal strength which has been observed by others (Graven-Nielsen et al. 1997b, 2002; Slater et al. 2003; Khan et al. 2011) but also represents the accentuation in the development of fatigue in HYP compared to CTRL. No difference in end-exercise MVF was observed, similar to Smith et al. (2020), despite a marked reduction in exercise time. This is reflected by the significantly greater ΔMVF/ΔTime in HYP compared to CTRL. It is likely that the force-generating capacity is reduced to a level which is associated with an inability of the participant to maintain sufficient neural drive to maintain target force, in line with the theory of the sensory tolerance limit (Hureau et al. 2018). During the earlier parts of the TTF, the reduction in MVF likely reflects a net inhibition of the motor unit pool which are used to generate knee extensor forces. Consequently, participants were exercising at the same absolute intensity, but a greater relative exercise intensity in HYP compared to CTRL.

In combination with measures of MVF was the delivery of peripheral nerve stimuli during and after each MVC which allowed for the quantification of central and peripheral fatigue during exercise. Voluntary activation, a measure of central fatigue, was significantly lower in HYP compared to CTRL at minute 1 which demonstrates the centrally mediated reduction in maximum force. No difference was observed at minute 2 which is unexpected because the maximum force was reduced at this time point. It is plausible that the interpolated twitch technique which was used to calculate voluntary activation may be insensitive to detect changes near maximal contraction intensities (Herbert and Gandevia 1999). As pain from the saline would have started to decrease in some individuals in tandem with an increase in naturally occurring exercise-induced pain, the inhibitory effect of pain may have been more difficult to capture with the ITT at minute 2 compared to minute 1. Furthermore, peripheral fatigue was not responsible for the change in MVF as the amplitude of the potentiated doublet remained unchanged between conditions, suggesting that the hypertonic saline had no impact on excitation–contraction coupling processes.

An interesting and novel finding within the present study is that the difference in the mean pain over the first minute of exercise between CTRL and HYP had a strong negative correlation with the difference in the change in MVF from baseline to minute 1 (*r* = − 0.859, *P* = 0.001) (Fig. 5). This was also the case for voluntary activation (*r* = − 0.773, *P* = 0.013), therefore providing strong evidence that central fatigue is mediated by the magnitude of pain perception and that pain may act in a ‘dose–response’ effect to cause central inhibition. In the present study, it was not possible to discern whether this effect is originating from the magnitude of the nociceptive signal or whether it is the conscious perception of the pain mediating this response, but future work could investigate this phenomenon.

### Electromyographic responses

There was a reduction in EMG amplitude at minute 1 and minute 2 in HYP compared to CTRL for both vasti muscles. This is in agreement with previous experimentally induced pain research (Graven-Nielsen et al. 1997b; Rice et al. 2019). A reduction in EMG amplitude may reflect a reduction in maximal central motor output to the quadriceps, that is likely centrally mediated. However, EMG amplitude may only provide a crude measure of neural drive (Farina et al. 2010) and it is likely that the reduced amplitude is an artefact of a reduction in force, as these two variables tend to scale linearly (Alkner et al. 2000; Campy et al. 2009). Nevertheless, a reduction in force/EMG without a change in doublet or M-Wave amplitude strengthens the notion that the reduction in force is centrally mediated. The bipolar EMG setup precludes the ability to identify which specific neural mechanisms are responsible for this, although previous work using fine-wire intramuscular EMG or high-density surface electromyography (HDEMG) during muscle pain may provide useful insight (Farina et al. 2004; Tucker et al. 2009; Martinez-Valdes et al. 2020). A reduction in motor unit firing frequency has previously been observed (Farina et al. 2004; Tucker et al. 2009) along with a de-recruitment of low-threshold motor units (Martinez-Valdes et al. 2020). Therefore, the reduction in force and EMG amplitude seen in this study may be due to a centrally mediated inhibition of motor units and/or a decrease in firing frequency in some of the motor units across the motor-neuron pool.

Interestingly, no difference was observed between conditions for submaximal EMG amplitude at minute 1 (when hypertonic saline pain was likely evoking the peak-pain response) (Fig. 4). It could have been expected that the earlier recruitment of higher threshold motor units to compensate for the pain mediated central inhibition and consequent acceleration of fatigue would have led to a greater increase in the EMG amplitude with HYP. However as previously seen, changes to motor unit firings rates and recruitment thresholds have been found without a concomitant change to the surface EMG amplitude (Martinez-Valdes et al. 2020). It is likely that a combination of excitatory and inhibitory processes occur in response to pain (Hodges and Tucker 2011) and during exercise the task, the demands can be maintained but at the cost of accelerated fatigability. Therefore, complex adjustments to motor control may not be detectable by a bipolar surface configuration. Furthermore, evidence suggests that muscle pain may result in a shift in the centre of gravity of activation (Liew et al. 2019) meaning that regional variations in muscle activity may occur, potentially outside of the detection volume of the bipolar configuration. However, it is not known if a similar change occurs with more widespread naturally occurring exercise-induced pain as opposed to the more localised muscle pain with hypertonic saline. At the point of task failure, there was a lower EMG amplitude in the VL but not the VM in HYP compared to CTRL. The reduced EMG amplitude is likely a reflection of the shorter TTF and an inability for the individual to rectuit as many high-threshold motor units in HYP which was necessary to prolong exercise time.

### TMS responses

TMS was delivered during the TTF to determine corticospinal excitability and inhibition in the presence of elevated muscle pain. First, corticospinal excitability was not different between conditions at any time point and also did not change over time. Discrepancies in motor cortex excitability in response to acute pain have been observed, with both a decrease (Le Pera et al. 2001) and increase (Rice et al. 2015) in excitability, whereas fatigue from a 2 min MVC also increased MEP area but was unchanged with the maintenance of group III/IV afferent firing (Kennedy et al. 2016). Differences in motor cortical excitability may be related to the level of muscle activity present during TMS delivery. MEPs evoked at rest appear to show a reduction in corticospinal excitability but not when delivered during an active contraction (Burns et al. 2016). Nevertheless, it appears that a reduction in corticospinal excitability was not responsible for the impaired endurance performance with elevated pain. On the other hand, corticospinal inhibition assessed with the TMS silent period increased over time and was greater at 100 s in HYP compared to CTRL, but not 10 s or at task failure. The lack of difference between conditions at 10 s is likely due to the lack of time for the saline to reach a level of pain which would cause a measurable lengthening of the silent period as pain VAS within the beginning of exercise was not different between CTRL and HYP (Fig. 2).

The silent period is thought to reflect activity of the gamma-aminobutyric acid b neurotransmitter which may be acting to inhibit the motor cortical activity, thus potentially impacting motor control and descending drive of the quadriceps during the TTF. Additionally, lengthening of the TMS silent period can be caused by changes at the spinal level which could be elucidated by cervicomedullary evoked potentials. Exercise-induced pain or fatigue may therefore also act to inhibit spinal motoneurones (Goodall et al. 2018; Škarabot et al. 2019). Consistent with these findings, Hilty et al. (2011) found that partial blockade of group III/IV afferents (including nociceptors) attenuated the lengthening of the silent period during exercise. In combination, these findings suggest that pain or an increased nociceptive firing acts to inhibit the corticospinal pathway and inhibit descending central drive to the quadriceps.

### Task disengagement versus fatigue

One potential mechanism of how pain may have reduced endurance performance relates to the aversiveness of pain due to the enhanced negative affective-motivational component associated with the hypertonic saline injection combined with the intense exercise. This potentially contributes to an increased avoidance drive to escape the pain from the endurance task (Navratilova and Porreca 2014; Stevens et al. 2018), and in this study, participants ended the exercise at a similar, potentially intolerable level of exercise-induced pain. However, end-exercise MVF and doublet amplitude were similar and a premature withdrawal from exercise would have likely resulted in less end-exercise fatigue. Furthermore, no difference in pain catastrophizing was seen between conditions which is associated with exercise performance and task disengagement (Nijs et al. 2008). It is plausible that a voluntary task disengagement did occur, but this effect was ‘masked’ by the exacerbation of neuromuscular fatigue. Furthermore, this ‘voluntary disengagement’ effect may be more prevalent in whole-body, longer duration exercise, or in non-exercised muscle groups; however, this warrants further investigation. Collectively, whilst there is not sufficient evidence to rule out task disengagement under the present experimental conditions, the differences in neuromuscular measures suggest that in this form of exercise their impact is greater. We therefore contend that an amplification of central fatigue best explains the reduction in TTF in HYP.

### Methodological considerations

Two females took part in the study, however, we did not control for what phase they were in of the menstrual cycle which may have altered their response to experimental pain (Sherman and LeResche 2006) potentially via ‘luteal analgesia’ (Vincent et al. 2018) and exercise performance/neuromuscular fatigue (Ansdell et al. 2019; McNulty et al. 2020). Future work should attempt to control for this factor.

The TMS stimulus intensity for the MEPs was determined by delivering stimuli during contractions at 20% of MVF to generate a stimulus–response curve. The lowest intensity to to evoke a maximal increase in the VL whilst minimising BF MEP was selected (Temesi et al. 2014). We acknowledge that by maximising the MEP amplitude there may be a potential for a ‘ceiling effect’ with MEP amplitude. In the present study, there was a main effect for time for MEP/M_max_ but subsequent post hoc tests revealed no differences between time points. It was possible that pain may have reduced the MEP but fatigue increased it, thus resulting in no net difference. Indeed, several other studies using the same stimulus intensity method have observed an increase in the MEP with fatigue (Pageaux et al. 2015; Kennedy et al. 2016; Aboodarda et al. 2020). Nevertheless, the utilisation of a stimulus intensity at 120% of active motor threshold may have provided greater sensetivity for increases in corticospinal excitability.

The hypertonic saline injected was 1 mL of 5.85% NaCl solution. Possibly due to differences in pain threshold and pain tolerance, there was a variable response in pain VAS to the hypertonic saline injection. One important consideration is those participants who did not report a significant increase in pain following the injection of hypertonic saline. For example, the difference in mean pain VAS scores when normalised for the same exercise time was on average 19 points greater in HYP compared to CTRL, whereas in three participants, it was as low as -4, 3 and 6 units, respectively. While this heterogeneous pain response may have allowed for a robust correlation analysis, some of the data in changes of neuromuscular parameters between CTRL and HYP may have become ‘diluted’ with these low responding participants. Indeed, work by Graven-Nielsen and colleagues (Graven-Nielsen et al. 1997a) demonstrated some individuals only rated peak pain as 1 cm on a pain VAS, whereas others were around 5–6 cm (out of 10 cm). Future work may want to take an individualised approach with injection volume to evoke a consistent pain response equal to ‘strong’ (~ 5/10 pain VAS).

## Conclusion

In summary, elevated muscle pain reduces strength and endurance performance due to centrally mediated mechanisms. It is likely that feedback from group III/IV afferent nociceptors is responsible for constraining motor output to the painful muscle group. A re-distribution/re-organisation of motor control may also be acting to maintain the demands of the isometric TTF but in a manner that causes fatigue to occur more rapidly.

## Data Availability

Raw data are available upon request from the corresponding author.

## Abbreviations

ANOVA: Analysis of variance
BF: Biceps femoris
CTRL: Control condition
EMG: Electromyography
HYP: Hypertonic saline condition
MEP_AMP_: Motor-evoked potential amplitude
MVC: Maximum voluntary contraction
MVF: Maximim voluntary force
PANAS: Positvie and negative affect schedule
RMS: Root mean square
RPE: Rating of perceived exertion
TMS: Transcranial magnetic stimulation
TTF: Time to task failure
VAS: Visual analogue scale
VL: Vastus lateralis
VM: Vastus medialis
